# The Current Status and Future Direction of Clinical Research in Japan From a Regulatory Perspective

**DOI:** 10.3389/fmed.2021.816921

**Published:** 2022-01-13

**Authors:** Hideki Maeda

**Affiliations:** Department of Regulatory Science, Faculty of Pharmacy, Meiji Pharmaceutical University, Kiyose, Japan

**Keywords:** clinical research, clinical trial, Japan, regulation, regulatory science

## Abstract

In Japan, a law called the Clinical Trials Act went into being effective on April 1, 2018, and clinical research on human subjects conducted in Japan has been undergone major changes. Those other than clinical trials for marketing approval of drugs or medical devices are broadly classified into “specific clinical trials” and others, and regulations have been tightened for each. As a result, clinical interventional study was drastically reduced, and observational clinical study increased. For the observational clinical study, the two previous ethical guidelines were merged into the “Ethical Guidelines for Medical and Biological Research Involving Human Subjects,” which was enacted in March 2021. The observational clinical study is now subjected to these ethical guidelines. In addition, changes are planned for the Act on the Protection of Personal Information, which greatly affects data collection in clinical research. Clinical research in Japan must be conducted appropriately while adapting to these various changes in the external environment and legal framework. Adapting to these changes is not an easy task, as it requires increased financial and human resources for all stakeholders.

## Introduction

Advances and developments in medical technology lead to higher quality medical care and better health for people. The creation and reinforcement of evidence based on clinical research are important for the development of medicine. In spite of this, clinical research in Japan is insufficient in terms of the related systems and implementation mechanisms and has therefore fallen behind Europe and the United States (1, 2). After the Diovan scandal, a misconduct case related to a post-marketing clinical trial of an antihypertensive agent, valsartan in 2012 and similar scandals involving the clinical research at that time (3), trust in the clinical research conducted in Japan was lost (4, 5). Since then, to regain trust in clinical research, industries, government, and academia have been united in their efforts to ensure the reliability and scientific soundness of clinical research, improve the mechanisms used to implement research, and create and revise laws and other regulations that support these changes. Against this background, in recent years, legal measures and policies related to clinical research are being strengthened in Japan.

The legal system concerning clinical research in Japan consists mainly of two laws or guidelines. One is the Clinical Trials Act (“*Rinsho-Kenkyuu hou*” in Japanese) for interventional research (6), which was established in April 2018. The other is an ethical guideline for medical research, such as observational clinical studies. This guideline is known as the Ethical Guidelines for Medical and Biological Research Involving Human Subjects (9), which was developed by merging the existing Ethical Guidelines for Medical Research Involving Human Subjects (7) and the Ethical Guidelines for Human Genome/Analysis Research (8). The new merged guideline was announced in March 2021. The Clinical Trials Act for interventional research was established in April 2018, and over 3 years have passed since its establishment. Although stakeholders such as researchers, medical institutions, and pharmaceutical companies that conduct interventional research are required to understand and appropriately comply with this Act, it is believed that there is still room for making further improvements in the Act. As the Clinical Trials Act was originally created for purpose of restoring trust in the clinical research conducted in Japan after several scandals, it requires bigger changes and more careful handling to be carried out by stakeholders, such as medical institutions and pharmaceutical companies than those required under the regulations stipulated by the existing ethical guidelines. While these changes were appropriate in some cases, in others, they simply led to increase in paperwork and complexity. The enactment of the Clinical Trials Act has caused continuing confusion at institutions where research is conducted; however, in general, it has led to the reduction of outdated habits, changes in ways of thinking, and improvements in clinical research operations as well as in the relationship between pharmaceutical companies and medical institutions. Based on this, I believe that the Clinical Trials Act currently remains effective in improving the clinical research conducted in Japan. It has been <1 year since the establishment of the Ethical Guidelines for Medical and Biological Research Involving Human Subjects, which targets clinical research other than interventional research, such as observational clinical studies. It can be expected that issues related to the handling of these guidelines will be brought up in the future, but the issue related to the definition of “observational clinical studies” has already been pointed out as a problem. Therefore, researchers, medical institutions, and pharmaceutical companies will search for better ways to carry out the clinical research in Japan.

In this paper, we provide an overview of the history of legal regulations related to clinical research and discuss the responsibilities and roles played by various stakeholders in Japan. The objective is to point out the current issues in the legal system and guidelines related to the Japanese clinical research and discuss the future direction of clinical research in Japan.

1. The types of clinical research

Clinical research is a part of medical research that is conducted for determining the causes and treatment of diseases; making improvements for disease prevention, diagnosis, and treatment; and improving the quality of life of patients. Clinical research naturally involves human subjects. There are a variety of definitions of “clinical research” and none of them has become the established definition; however, it is believed that clinical research can be classified into the following four types:

(1) Clinical interventional study: research related to the development of medicines, treatments, therapeutic methods, and drugs.
(2) Prognostic factor clinical study: research that investigates factors that predict patient prognoses.
(3) Epidemiological clinical study: research that investigates the causes of diseases.
(4) Validity clinical study: known as a validation study, this research assesses tests and surveys.

Prospective clinical research includes interventional studies involving interventions, such as drugs; medical devices; surgery; radiation, exercise, and diet therapies as well as non-interventional studies or observational studies, which do not involve any intervention. Specially in Japan, prospective clinical research conducted for obtaining approval to manufacture and market drugs and medical devices is known as a “clinical trial for the approval of drugs or medical devices” (“*Chiken*” in Japanese). *Chiken* fall under the regulations of Japanese Good Clinical Practice (J-GCP) which is more stringent guideline than international guideline for GCP (ICH-GCP). As a result of the establishment of the Clinical Trials Act in 2018, clinical research that involved interventions other than *Chiken* and was conducted under previously existing ethical guidelines that also need to comply with the new law.

2. Legal regulations related to clinical research

In Japan, the first legal regulation related to clinical research other than *Chiken* consisted of guidance in the form of ethical guidelines for each type of study, i.e., observational clinical study, clinical research, and human genome/analysis research.

The first regulation was the Ethical Guidelines for Human Genome/Analysis Research (8) developed in 2001. In addition, the Ethical Guidelines for Epidemiological Clinical Study (10), which targeted observational clinical studies conducted in the field of epidemiology, was developed in June 2002. The Ethical Guidelines for Clinical Research (11) was developed in 2003 and covered clinical research other than those mentioned above. Thus, each type of clinical research was conducted in accordance with one of the above ethical guidelines. Subsequently, from around 2011, problems, such as overlapping guidelines and uncertainties regarding the guideline that should be followed when conducting research that would fall under multiple ethical guidelines were brought up. Further, in the wake of the 2012 Diovan incident (3), a review of ethical guidelines was conducted; in December 2014, the Ethical Guidelines for Epidemiological Clinical Study (10) and the Ethical Guidelines for Clinical Research (11) were merged, and the Ethical Guidelines for Medical Research Involving Human Subjects (7) was officially announced. Based on what was learned as a result of the Diovan scandal, the legal system and financial aspects were reviewed, which led to the enactment of the Clinical Trials Act for interventional research in April 2018 (6). In addition, the Ethical Guidelines for Medical Research Involving Human Subjects (7) and the Ethical Guidelines for Human Genome/Analysis Research (8) were merged, and the Ethical Guidelines for Medical and Biological Research Involving Human Subjects (9) was established in March 2021. Therefore, currently, the clinical interventional research that receives funding from a company and similar studies fall under the Clinical Trials Act (6) and all other clinical research falls under the Ethical Guidelines for Medical and Biological Research Involving Human Subjects (9).

**Figure 1:**
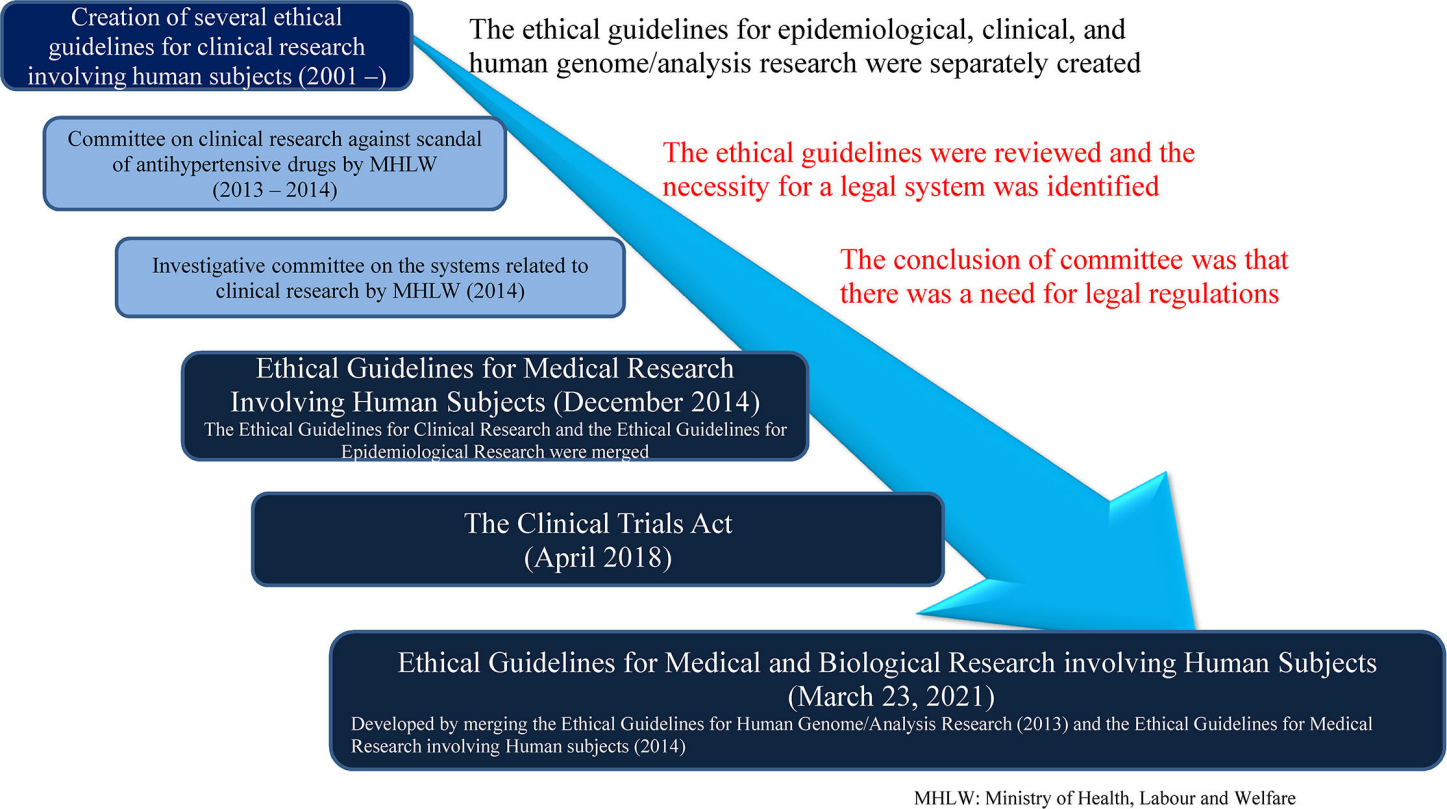
Major steps for regulations and guidelines related to clinical research in Japan.

The main changes that the legal regulations related to clinical research have undergone are shown in Figure 1.

“Clinical research” as defined by the Clinical Trials Act is interventional research other than *Chiken* designed to identify the efficacy or safety of drugs and other products through the use of drugs, medical devices, etc., by people. The Clinical Trials Act defines “specific clinical trials” (“*Tokutei-Rinsho-Kenkyu*” in Japanese) as interventional trials on previously approved drugs and medical devices that receive funding from companies, clinical interventional studies on unapproved drugs and medical devices, and interventional clinical research for off-label uses. Specific clinical trials must be conducted in accordance with the Clinical Trials Act, and medical institutions must have a research system and all relevant standards established. In addition, when conducting specific clinical trials, a Certified Review Board (CRB) is required to inspect and approve the study, and the study protocol must be submitted to the Ministry of Health, Labor, and Welfare. As of July 1, 2021, 101 medical institutions have CRBs. The medical institution or institutions conducting the research and all researchers involved in the research must reveal any conflict of interest (COI) related to the financial support received from pharmaceutical companies. Prior to the enactment of the Clinical Trials Act, it was not necessary in Japan to have an established research system, obtain CRB approval after due inspection, reveal COI, or submit any paperwork to the Ministry of Health, Labor, and Welfare.

3. Implementation scheme and role allotment

The Clinical Trials Act assumes that the research initiative is conducted by either a researcher or a researcher in cooperation with a company. On the other hand, in cases of clinical research on already approved drugs and medical devices that is conducted by companies as post-marketing clinical trials or surveillance, the research must be conducted in accordance with a risk management plan (RMP) and the company must conduct the research as the sponsor in Japan. Thus, based on the research initiative, interventional clinical research in Japan is currently carried out as one of the following three types:

(1) Investigator-initiated research.
(2) Joint research with company (investigator-initiated).
(3) Joint research with company (company-initiated).

As there must be a particular format for administrative procedures and contracts, which are required during study implementation, the Japan Pharmaceutical Industry Legal Affairs Association (*Ihoken*) has established formats for contracts and other documents used in each type of clinical research (12).

The research material, labor, and financial support that companies may provide for conducting clinical research under the Clinical Trials Act are detailed in Table 1. Regardless of the type of clinical research, there are precautions stipulating that companies cannot be involved in the selection of participating centers; execution of any tasks related to applying for the approval of Institutional Review Boards; submission of study protocols to the Ministry of Health, Labor, and Welfare; and execution of activities, such as monitoring, supervision, data management, or statistical analysis (13). Furthermore, while there are guidelines related to financial support provided by companies for clinical research that is not covered by the Clinical Trials Act, there are currently no clear guidelines on the contents and labor that companies can provide as support.

**Table 1:** The involvement of companies under the Clinical Trials Act in Japan.

		**Investigator-initiated clinical research**	**Joint clinical research**
		**Investigator-initiated**	**Industry-initiated**
Companies may	– Post calls for research proposals on the Web or other procedures – Provide research funding under research contract – Conduct feasibility check for a study – Request progress and result reports from the investigator as per the contract – Request termination of the contract and return of research funding in cases in which the progress of the study is markedly delayed – Conduct prior review of items scheduled to be publicly announced	– Provide research funding under research contract – Conduct feasibility check for a study – Be involved in creating the study protocol – Be involved in creating the statistical analysis protocol – Perform special analysis, etc. as a part of a study, etc. and provide a result report for that analysis – Request progress and result reports from the investigator as per the contract – Request termination of the contract and return of research funding in cases in which the progress of the study is markedly delayed – Participate in meetings with the investigators	– Provide research funding under research contract – Conduct feasibility check for a study – Create the study protocol – Create the statistical analysis protocol – Perform special analysis, etc. as a part of a study, etc. and provide a result report for that analysis – Request progress and result reports per the contract – Request termination of the contract and return of research funding in cases in which the progress of the study is markedly delayed – Participate in meetings with the investigator(s) – Write the paper
Companies may not	– Be involved in selection of the participating investigational sites – Request for research proposals from the investigators – Perform statistical analysis-related tasks – Be involved in the analysis and discussion of the research results – Participate in meetings with the investigators	– Select the participating investigational sites – Request review to Certified Review Board – Submit notification of study protocol to the Ministry of Health, Labour and Welfare – Monitoring and inspection – Conduct data management – Conduct statistical analysis – Medical writing of reports – Write the paper	– Select the participating investigational sites – Request review to Certified Review Board – Submit notification of study protocol to the Ministry of Health, Labour and Welfare – Monitoring and inspection – Conduct data management – Conduct statistical analysis

4. Current issues with the Clinical Trials Act

Although it has been a little over 3 years since the enactment of the Clinical Trials Act, several issues related to the implementation have been pointed out (14). Comparison of the Clinical Trials Act to the regulations stipulated by the previous ethical guidelines reveals a number of characteristic features. Examples are listed below:

- The new category of clinical research known as “specific clinical trials.”
- The establishment of CRBs, which allows centralized inspection rather than inspections at each center.
- The shift in the responsibility of the research from the director of the center to the principal investigator (researcher).
- The establishment of details regarding conflicts of interest.

A specific clinical trial is a clinical research that satisfies at least one of the following: (1) Utilizes research funding provided by the manufacturer and marketer of the drug for which the research is being conducted, and (2) Utilizes drugs that are either unapproved or are being used off-label. However, as clinical settings are complex, there are a variety of questions regarding the exact moment that a clinical trial begins. For example:

- Is a clinical trial with dose modifications for elderly or children that are common in routine practice but strictly off-label considered as a “specific clinical trial”?
- Are studies utilizing an old drug that is covered by insurance for an off-label purpose considered “specific clinical trials”?
- Is it acceptable to not classify as a “specific clinical trial” an “observational clinical study” whose funding is provided by a pharmaceutical company in cases in which testing is not performed during standard medical examinations or when a higher number of examinations and tests are performed than would be as a part of standard medical examinations?
- There are no issues on the study drug of the anticancer drugs used in the study, but if the research funding is provided by the company of the antiemetic agent used in the study, is it acceptable to exclude from the “specific clinical trial”?

In addition, there are no clear guidelines regarding rules and the allotment of responsibilities, which make it difficult to know how to handle such issues.

For example, there is no single uniform way to make judgments in cases wherein it would be better to obtain the consent of the study participants for the purpose of having a paper published by a leading journal. However, according to the ethical guidelines, patients can opt out of granting consent to participate in studies in which the methods for gathering and reporting safety information, as required for observational clinical studies, are not established or in cases in which the requirements of the principal investigator and medical institutions implementing the study are not clear. There are also cases in which the monitor conducts an excessive amount of source data verification. Finally, there are examples in which companies are still involved in the creation of protocols, selection of centers, analysis, and case investigation even though they are prohibited from doing so.

5. Current issues, future direction, and effort toward revising the Clinical Trials Act

As little time has passed since the establishment of the Ethical Guidelines for Medical and Biological Research Involving Human Subjects, the issues with these guidelines have yet to be clearly identified. However, 3 years have passed since the Clinical Trials Act has been established, and discussions on how to improve it are currently under way. Here, we will list several points of dispute regarding the revision of the Clinical Trials Act, its current state, and our opinion regarding the direction that the improvements should take.

(1) The handling of observational clinical studies
**Current Status**
Although observational clinical studies are not subject to the Clinical Trials Act, the definition of an observational clinical study is not clear; therefore, there are cases that should not necessarily be excluded from the regulations of the Act simply because the researcher calls their study as an “observational clinical study.” In particular, there are cases in which actions, such as additional hospital visits for the purpose of the study, the addition of measurement items, and collection of small amounts of additional blood sampling are determined not to be “the most appropriate medical care for the patient” and, as a result, the CRB determines that the study should be classified as a specific clinical trial.
**Making Improvements**
- The scope of application needs to clearly indicate “interventional studies that utilize drugs, etc.”
- The definition of “observational clinical studies,” which are excluded from the Act, needs to be revised.
(2) The concept of “sponsor”
**Current Status**
The principal investigators and all centers that are involved in study implementation play the role of both a “sponsor” and an “investigator.”
**Making Improvements**
- Each study should have one sponsor.
- Sponsors can be individuals, companies, research institutions, or organizations.
- Sponsors are responsible for the implementation of the study (e.g., regarding adverse event reports, it should be determined by the sponsor whether there is a causal relationship with test drugs or not, and based on adverse event reports collected from the participating investigators).
(3) CRB requirements
**Current Status**
There are many CRBs in Japan, and there exists a wide disparity in the review standards, procedures, skills, and fees, which means that there are cases in which the appropriate review is not conducted.
**Making Improvements**
Based on the fact that there are disparities in the quality of CRBs, in the future, the CRBs should be consolidated.
(4) The scope of applying the Clinical Trials Act in studies involving medical devices
**Current Status**
“Off-label” refers to cases of usage that differs even slightly from the approved, certified, or applied for usage, efficacy, and performance. If “off-label,” then the study is subject to *Chiken* or the Clinical Trials Act.
**Making Improvements**
With respect to the clinical research involving off-label medical devices, cases in which the medical device can be regarded as having the same level of risk as that determined when the medical device in question received certification, the status of the study should be investigated and the issue of whether the study should be subjected to *Chiken* or the Clinical Trials Act based on the results of that investigation should be considered.

## Discussion and Conclusion

In the wake of scandals involving clinical research, such as the 2012 Diovan scandal, efforts have been under way to ensure the trustworthiness and scientific soundness of clinical research, strengthen regulations and guidelines for clinical research, and examine and adjust the regulations that support these changes to regain trust in the Japanese clinical research. Against this background, in recent years, legal measures and policies related to clinical research have been taken in succession in Japan. The Japanese medical institutions, pharmaceutical companies, and stakeholders in regulatory agency must carry out clinical research appropriately while adapting to a variety of external environment-related and legal changes. Handling these changes will not be easy as they entail increases in funding and human resources. However, currently, clinical research in Japan is undergoing major changes and working toward improvements. We expect that as a result of these improvements, the Japanese clinical research will develop further and make additional contributions toward medical progress.

## Data Availability Statement

The original contributions presented in the study are included in the article/supplementary material, further inquiries can be directed to the corresponding author/s.

## Author Contributions

The author confirms being the sole contributor of this work and has approved it for publication.

## Conflict of Interest

The author declares that the research was conducted in the absence of any commercial or financial relationships that could be construed as a potential conflict of interest.

## Publisher's Note

All claims expressed in this article are solely those of the authors and do not necessarily represent those of their affiliated organizations, or those of the publisher, the editors and the reviewers. Any product that may be evaluated in this article, or claim that may be made by its manufacturer, is not guaranteed or endorsed by the publisher.
